# Integrated care in ovarian cancer “IgV Ovar”: results of a German pilot for higher quality in treatment of ovarian cancer

**DOI:** 10.1007/s00432-015-2055-6

**Published:** 2015-10-23

**Authors:** M.-D. Keyver-Paik, A. Abramian, C. Domröse, A. Döser, T. Höller, M. Friedrich, W. Meier, K. Menn, W. Kuhn

**Affiliations:** Department of Gynecology and Obstetrics, Center for Integrated Oncology Cologne Bonn, University of Bonn, Bonn, Germany; Department for Biostatistics, University of Bonn, Bonn, Germany; Department of Gynecology and Obstetrics, Helios Klinikum Krefeld, Krefeld, Germany; Department of Gynecology and Obstetrics, Evangelisches Krankenhaus Düsseldorf, Düsseldorf, Germany; Barmer GEK Nordrhein-Westfalen, Düsseldorf, Germany

**Keywords:** Ovarian cancer, High-volume hospitals, Quality, Cytoreduction, Survival

## Abstract

**Introduction:**

Late-stage ovarian cancer patient’s survival depends on complete cytoreduction and chemotherapy. Complete cytoreduction is more often achieved in institutions with a case volume of >20 cases per year. The Integrated care program Ovar (IgV Ovar) was founded in 2005 and started recruiting in 2006 with 21 health insurances and six expert centers of ovarian cancer treatment as a quality initiative. Results of the pilot and outcomes of patients of three participating centers will be presented here.

**Methods:**

Data of 1038 patients with ovarian cancer were collected. Adjuvant patients (*n* = 505) stage FIGO IIB-IV (*n* = 307) were analyzed for cytoreduction and survival. FIGO IIIC patients were analyzed separately.

**Results:**

Median follow-up was 32.7 months. Progression-free survival (PFS) was 23.1 months and overall survival (OS) was 53.6 months for stage IIB-IV. Patients with FIGO IIIC were completely cytoreduced in 48 %. PFS was 21, 29 months if completely cytoreduced. OS was 47.4, 64.9 months if completely cytoreduced.

**Discussion:**

Although the IgV Ovar Rhineland proved to have some structural problems with recruitment and prospective data collection, cytoreduction rates and outcome of patients prove treatment of patients in expert centers is superior to the national and international mean. Therefore, a new quality initiative will be started to bring more awareness to women and to their gynecologists and general practitioners of just how important a good referral strategy is.

## Introduction

 With 75 % ovarian cancer is still typically diagnosed at late stages, at this point, peritoneal carcinosis has most often spread to all quarters of the abdomen and/or to the pleural surfaces (FIGO IIIC or IVA). These women have a poor prognosis despite all efforts and have a 5-year survival rate of <25 %. Over three quarters of these patients present with serous papillary histology (Heintz et al. 2006).

Therapy of the disease is a combination of surgery and chemotherapy. Agents used for chemotherapy have been platinum, usually carboplatin, and a taxane, most often paclitaxel. Despite a few changes in regimen or (internationally) in sequence and route of application from intravenous to adding intraperitoneal, this has not changed for more than a decade (du Bois et al. 2003, 2010; Armstrong et al. 2006; Polcher et al. 2009; Pignata et al. 2014).

Prognostic factors are age, performance status, stage, histologic type, grading, pN1 status and the completeness of cytoreduction (Chi et al. 2001). Whereas the first six parameters are inherent, cytoreduction is influenced not only by the sites of the metastatic peritoneal tumor but greatly by the abilities of the individual surgeon and by the infrastructure available at the hospital (Kuhn et al. 2001; Crawford et al. 2005; Chi et al. 2006).

Researchers, mainly from the USA, but also from Germany, have shown treatment in hospitals with an interdisciplinary approach of the gynecologic oncologist as a surgeon with visceral surgeons and urologists improves the outcome of patients with ovarian cancer (Wimberger et al. 2007; Bristow et al. 2009). Equally important to the structure and case volume of the hospital is the experience and case volume of the operating gynecological surgeon (du Bois et al. 2009; Bristow et al. 2014).

Unfortunately, besides these well-published and researched facts, in Germany treatment of patients with late-stage ovarian cancer is not restricted to specialized centers. Basically, any surgeon’s belief in his or her own abilities qualifies for the undertaking of treatment of these patients, in some cases producing surgical outcomes of low quality with impaired survival of the patient.

In 2005, by initiative of the public health insurances, specialized gynecologic oncologists from six centers for treatment of ovarian cancer in the Rhineland came together with 21 health insurers forming the idea of an integrated care program (IgV) for patients with ovarian cancer.

The program was implemented to increase the amount of patients being treated in hospitals with high quality of surgical performance and in hospitals with adequate infrastructure, therefore increasing the rate of successfully completely cytoreduced patients.

Results from the “Integrated care in ovarian cancer (“IgV Ovar”) Rhineland” and outcomes of patients from three participating hospitals will be presented here.

## Methods

Criteria of quality were developed with international specialists of the field. They were laid down in a contract with the health insurance companies for hospitals wanting to take part in the initiative. These included structural criteria of the clinic as a whole for measurement of the interdisciplinary work with visceral surgeons and urologists, structural quality criteria for the gynecologic department for insuring operations by board-certified gynecologic surgeons, and number of carried out operations on patients with ovarian cancer in the last years per department and per surgeon and for chemotherapy application.

After diagnosis of ovarian cancer, each patient insured with any of the participating health insurances was asked whether she would like to participate in this quality initiative. If the patient was interested and registered per informed consent, the referring gynecologist was asked whether he/she was willing to cooperate, integrating the practice into the after-treatment clinical follow-up, with the patient presenting at the clinic and the referring gynecologist one time over the other.

Similar to other integrated programs (e.g., DMP Mamma), the referring gynecologist was asked to register with the initiative and would receive a fee for completing some follow-up forms on seeing the patients. Patients were to answer some Quality of life (QoL) questionnaires on presenting for clinical follow-up. All generated data were to be sent to the individual health insurance of the patient per fax.

To prove the idea, treatment of ovarian cancer patients in centers of experience would lead to better outcome of these patients, and to prove the principal idea of a centralized care for these patients, three of the participating hospital agreed to pool their data for treatment of patients with all stages of ovarian cancer in their institutions. To analyze quality of treatment, focus was laid on adjuvant treated patients with advanced stages (FIGO IIB–IV) since these patients represent a homogenously treated group. More analysis was done for FIGO IIIC patients, for these are presenting with surgically challenging conditions.

Data of all patients, treated in the Department of Obstetrics and Gynecology of the Helios Klinikum in Krefeld, the Evangelisches Krankenhaus in Düsseldorf and the University Hospital in Bonn were collected retrospectively. Since all hospitals are certified by the Deutsche Krebsgesellschaft (DKG) as “Gynäkologische Krebszentren”, significant parts of the follow-up were available, and missing data were completed.

Statistical analysis was performed using the IBM program SPSS 22, IBM, Ehningen, Germany.

## Results

Data of all patients surgically treated with ovarian cancer in the three hospitals between 2006 and 2012, 1038 patients in total, were collected (see Table 1). Out of 505 patients with adjuvant treatment, 307 patients were treated with advanced disease (IIB–IV) used for this analysis (see Table 2).

**Table 1:** Collective of patients

Type of neoplasia	*n*
Borderline	152
Primary adjuvant	505
Primary neoadjuvant	127
Relapse	173
Other than EOC	81
Total	1038

**Table 2:** Patient characteristics and postoperative status

FIGO IIB–IV	
*Age*	
Mean	63.37 (24.96–99.93) years
*FIGO* (*IIB*–*IV*)	307
IIB–C	18 (5.9 %)
IIIA	8 (2.6 %)
IIIB	35 (11.4 %)
IIIC	198 (64.5 %)
IV	48 (15.6 %)
*ASA*	
1	18 (5.9 %)
2	187 (60.9 %)
3	73 (23.8 %)
n.a.	4 (1.3 %)
*Grading*	
1	2 (0.7 %)
2	108 (35.2 %)
3	195 (63.5 %)
n.a.	2 (0.7 %)
*Nodal status*	
pN0	66 (21.5 %)
pN1	118 (38.4 %)
pNx	102 (33.2 %)
n.a.	21 (6.8 %)
*Cytoreduction*	
Complete	155 (50.5 %)
≤1 cm	87 (28.3 %)
>1 cm	65 (21.2 %)

The median age was 63.4 years (range 25–100 years), 79 % presenting with FIGO III disease. ASA classification (grading of patients for surgical procedures of the American Society of Anesthesiologists) was used as surrogate marker for performance status and was ASA 2 in 61 % and ASA 3 in 24 % of cases. Twenty-four percentage of patients presented with Grade 3 tumors, and 38 % had lymph node involvement. Fifty percentage of patients underwent systematic lymphonodectomy, in which 9.5 % bulky nodes were removed. Median number of removed nodes was 38 (95 % CI 7–138). Median operation time was 290 min (95 % CI 29–695 min).

Fifty percentage of patients were cytoreduced to no residual tumor, and 28 % had residual tumor ≤1 cm (Table 2).

**Fig. 1:**
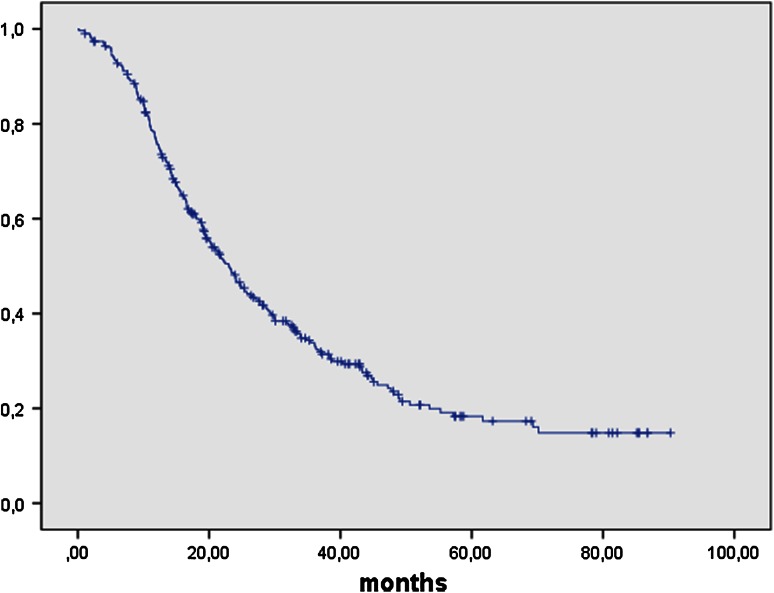
Progression free survival FIGO IIB-IV in months: PFS in 307 patients with FIGO IIB–IV ovarian cancer was 23.1 months (95 % CI 20.0–26.1 months)

**Fig. 2:**
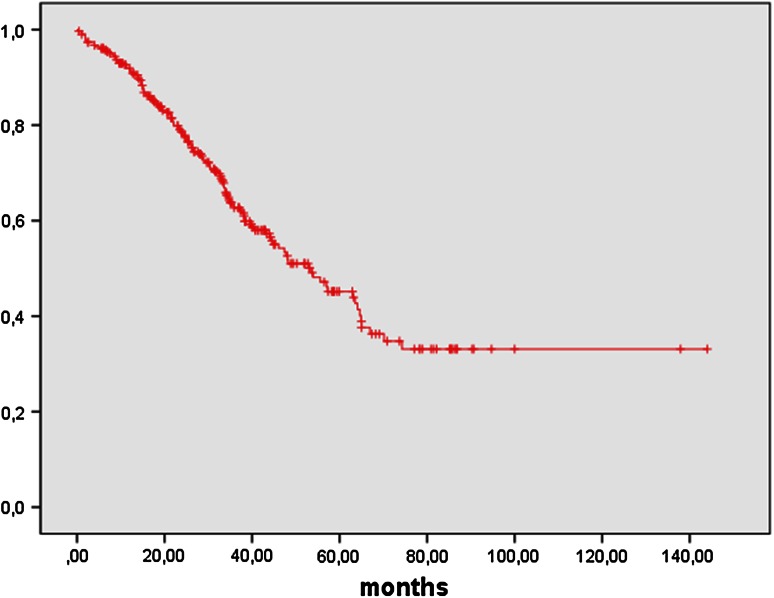
Overall survival FIGO IIB-IV in months: OS in 307 patients with FIGO IIB–IV ovarian cancer was 53.6 months (95 % CI 41.6–65.5 months)

Median follow-up was 32.7 months (95 % CI 0.5–100 months). Progression-free survival (PFS) was 23.1 months (95 % CI 20.0–26.1 months) (Fig. 1) and overall survival (OS) was 53.6 months (95 % CI 41.6–65.5 months) in patients with stage IIB–IV (Fig. 2). To this group of adjuvant patients, clinic A contributed 73 out of 156 patients with stage FIGO IIB–IV operated on during this time, clinic B 137 out of 156 and clinic C 97 out of 107. All three centers are therefore very much equal in their hospital volume for advanced ovarian cancer surgery, and survival time did not differ between the clinics (OS *p* = 0.963; PFS *p* = 0.327).

**Fig. 3:**
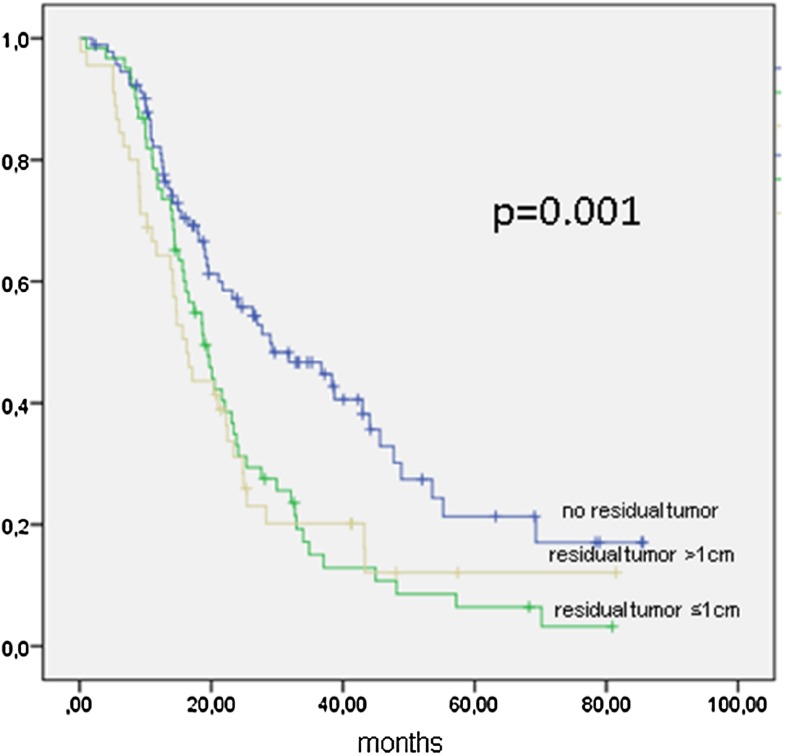
Kaplan–Meier function of progression-free survival in 198 patients with FIGO IIIC ovarian cancer stratified by cytoreduction

Patients with FIGO IIIC (*n* = 198) were completely cytoreduced in 92 cases (46.46 %), and 61 patients (30.81 %) were cytoreduced to ≤1 cm (Table 3). PFS was 21 months (95 % CI 18–24 months), 28 months if completely cytoreduced (95 % CI 16.2–41.7 months; *p* = 0.001), 18.9 months (95 % CI 15.2–22.5 months) if a residual tumor of ≤1 cm, 16.2 months (95 % CI 13.1–19.4 months) if tumor residuals of >1 cm remained (Fig. 3; Table 3).

**Table 3:** Progression-free survival in months of 198 patients with FIGO IIIC ovarian cancer stratified by cytoreduction

PFS	Cytoreduction	Median	
		Months	95 %-CI
FIGO IIIC	No residual tumor	28.977	16.241–41.714
	≤1 cm residual tumor	18.858	15.243–22.473
	>1 cm residual tumor	16.263	13.099–19.426
	All	21.027	18.027–24.026

**Table 4:** Overall survival in months of 198 patients with FIGO IIIC ovarian cancer stratified by cytoreduction

OS	Cytoreduction	Median	
		Months	95 %-CI
FIGO IIIC	No residual tumor	64.887	63.578–66.196
	≤1 cm residual tumor	34.070	28.920–39.220
	>1 cm residual tumor	34.760	20.120–49.400
	All	47.376	38.807–55.944

**Fig. 4:**
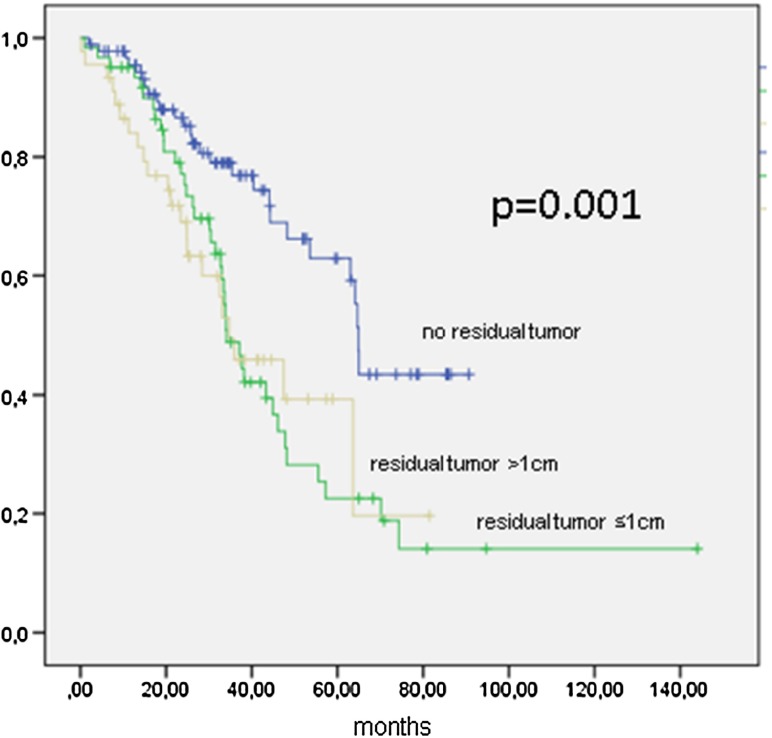
Kaplan–Meier function of overall survival in 198 patients with FIGO IIIC ovarian cancer stratified by cytoreduction

 OS was 47.4 months (95 % CI 38.8–55.9 months) and 64.9 months (95 % CI 63.6–66.2 months; *p* = 0.001) if completely cytoreduced, 34.1 months (95 % CI 28.9–39.2 months) if a residual tumor of ≤1 cm, and 34.8 months (20.1–49.4 months) if tumor residuals of >1 cm remained (Fig. 4; Table 4).

## Discussion

Pooled data analysis proved the selection criteria for participating hospitals successfully insured the desired improvement of quality of treatment for the patient.

**Fig. 5:**
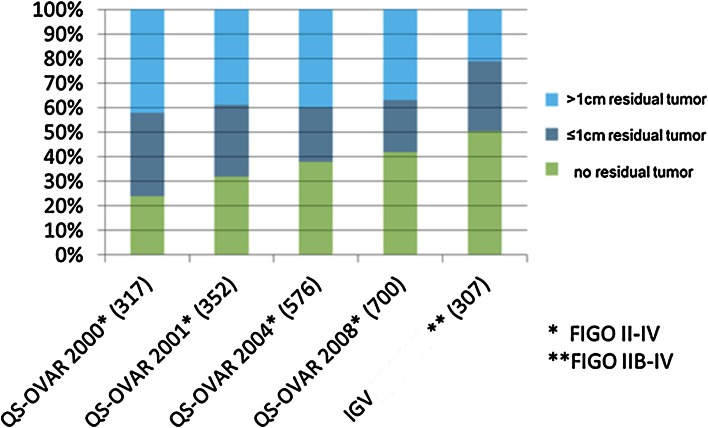
Cytoreduction: comparison QS-Ovar versus IgV hospitals. Modified after QS-Ovar, Hilpert et al. (2010)

In 2000, 2001, 2004 and 2009, the Arbeitsgemeinschaft für Gynäkologische Onkologie Ovar (AGO Ovar) published surveys on German hospitals for quality of treatment, using postoperative status of cytoreduction as the most important indicator (Hilpert F 2010). Over the surveyed years, the ratio of patients with FIGO II–IV with complete cytoreduction was improved from initially less than 25 % to about 41 % in the last survey. The participating IgV hospitals achieved a rate for complete cytoreduction of 51 % (Fig. 5).

The well-established Munich tumor register reports a 32 % rate of complete tumor resection for patients with stage FIGO IIIC disease (Schmalfeldt et al. 2014). The participating IgV hospitals achieved a rate of complete cytoreduction of 47 % in the same collective.

Data from FIGO IIIC patients show the importance of complete tumor resection (Figs. 3, 4). Residual tumor of any size will compromise the survival of the patient, since there is no statistical difference between gross residual tumor and tumor lesions under or over 1 cm (OS 34.1 vs. 34.8 months, n.s.). This result is to be expected and ties in with most published data (Chang and Bristow 2012; Keyver-Paik et al. 2013). The same effect of any residual tumor was seen for all other stages in the performed analysis outside FIGO IIIC (data not shown).

With high rates of complete cytoreduction, PFS and OS were 23.1 and 53.6 months, respectively, for patients with FIGO IIB–IV comparing to PFS and OS survival rates in literature of 18.2 and 44.1 months in a German collective of patients on chemotherapy trials (du Bois et al. 2009). International trial results, e.g., ICON7, show a PFS of 17.5 in the control and 19.9 in the research arm (+bevacizumab) and an OS of 30.3 versus 39.7 months for stages I–II high risk and IIB–IV (Perren et al. 2011; E Pujade-Lauraine 2014). In patients with stage FIGO IIIC, the three IgV hospitals achieved a PFS of 21 months for all patients and 29 months when no residual tumor was present. Overall survival was 47.4 months for all and 64.9 when no residual tumor was present. In ICON7, stage FIGO IIIC patients achieved a PFS of 17.5 versus 19.9 and an OS of 58.6 versus 58.0 months. Although retrospective data, the results of the three IgV hospitals are representing an excellent outcome for patients treated in these hospitals especially considering the data comes from a non-trial patient collective.

In 2009, du Bois et al. published a meta-analysis of surgical outcome comparing operations by board-certified gynecologic oncologists to other gynecologic surgeons or surgeons of other disciplines, e.g., visceral surgeons (du Bois et al. 2009). The data clearly was in favor of the specialized gynecologic oncologists, showing the knowledge of the disease and the acknowledgement of cytoreduction as the one most important and influenceable factor in ovarian cancer is the key to a successful operation.

Other published data are showing, when comparing surgical effort in experienced centers to less-experienced centers, less-experienced centers will significantly underperform (all *p* < 0.0001) in systematic lymphonodectomy, bowel surgery and specifically in peritoneal stripping. In this analysis, it is most discomforting to see, even basic measures, as hysterectomy and bilateral adnexectomy and omentectomy are significantly less frequently carried out in less-experienced centers (*p* < 0.0001) (Wimberger et al. 2007).

A data analysis of 20,600 patients in the USA, published in 2010, also proved a statistical correlation between case volume for FIGO IIIC and IV patients and survival (Bristow et al. 2010). A case volume of >20 cases per hospital showed a better overall survival for these patients. A later analysis of the same author of almost 12,000 patients defines high-volume hospitals as hospitals with >20 cases, and high-volume physicians as physicians with >10 cases (Bristow et al. 2014). Patients treated in high-volume hospitals by high-volume physicians have a statistically significant better survival than patients treated by surgeons with less cases in smaller hospital units. Any other combination is also less favorable than the high-volume combination.

These results were published well after the contracting for the IgV Ovar in 2006, but precisely underline the correctness of the quality strategy applied by the network.

Cost effectiveness was no parameter in the planning of the IgV Ovar but data from literature suggest a referral strategy to more experienced centers is also cost-effective. An American analysis from 2007 showed although treatment in experienced centers may be more expensive initially (39,957$ vs. 50,652$), when adjusted for QoL and survival time, experienced centers were the more cost-effective healthcare strategy (17,149$ vs. 9893$) (Bristow et al. 2007).

This analysis of outcome proves the hypothesis of bettering the treatment of patients in specialized centers, and it is probably also a cost-effective strategy, albeit there were also some problems in the pilot model of integrated care.

Data collection was not done to the standard aimed for at the beginning of this pilot. Quality of data on QoL proved insufficient for a comprehensive analysis.

After some thorough investigation into reasons given for the reported shortfalls in data collection and recruitment, the cardinal points are:

Referring gynecologists were more reserved to the program than expected: partly, since participation of their patients in this program meant more documentation on an occupation already filled with documentation on a daily basis, and partly because of a general distrust toward a data collection being forwarded to the participating health insurers.

On the side of the hospitals’ clinicians, identifying eligible patients for the program was also some obstacle to overcome because of the myriad of health insurances, in some cases with a very local structure, in the German health system. Only patients with the participating insurances were to be included in the program, and in some cases, although one local branch of an insurance company may take part, another local branch of the same insurance might not participate. This made it difficult to oversee for the clinician to which patient to offer the program without permanently carrying a list of the insurances. No private insurance did participate in the program.

A negative attitude toward more documentation was also one of the stumbling blocks for hospital clinicians. All hospitals are certified “Gynäkologische Krebszentren” and documentation and follow-up are already done. Hospitals employ staff for documentation of patients with gynecological cancer and their treatment and follow-up, but with no common electronic platform, and using an overcome system of paper and fax, double documentation of the same patient was necessary.

There was no regular central controlling mechanism until the end of the project in 2015. Therefore, regular surveillance on data collection and data quality was not established. The decentralized mode of data collection in the different clinics, private practice and health insurances helped to cover up the lack of complete documentation for some extended time.

While the contract was very specific on the quality assurance of the hospitals structure for treatment for patients, it was lacking to specify the quality of structure for documentation. It would have been advisable to install a case manager for patients on the program in every hospital.

Ovarian cancer still is a disease with a poor prognosis. Surgical outcome with complete cytoreduction is the one most important prognostic factor for the survival of a patient. Too many patients in Germany today are still not referred to centers of experience, where an interdisciplinary setup and an experienced and well-educated gynecological surgeon will see to the success of the cytoreduction. A suboptimal referral strategy still costs life and life-time even today in Germany.

The quality initiative “IgV Ovar” in the Rhineland was a project set up by some health insurances and initiating expert centers of the region and under the consultation of international experts in this field. IgV Ovar did successfully prove, treatment in expert centers leads to a better outcome of patients. Therefore, although not all expectations were met, a new initiative will be started soon based on the concluded pilot.

Avoiding double documentation and excessive administrative detail, we will approach hospitals to come together with the assistance of the health insurance companies to voluntarily publish their quality indicators of case volume and outcomes in a benchmarking system. In return, we will initiate an awareness campaign for patients, gynecologists and general practitioners publishing the benchmark and emphasizing the importance to be treated in expert centers when ovarian cancer is diagnosed.
